# The role of nocturnal delivery and delivery during the holiday period in Finland on obstetric anal sphincter rupture rates- a population based observational study

**DOI:** 10.1186/1756-0500-3-32

**Published:** 2010-02-05

**Authors:** Sari Räisänen, Katri Vehviläinen-Julkunen, Mika Gissler, Seppo Heinonen

**Affiliations:** 1 Department of Nursing Science, University of Eastern Finland, PO Box 1627, FI-70211 Kuopio, Finland; 2 Department of Obstetrics and Gynaecology, Kuopio University Hospital, PO Box 1777, FI-70211 Kuopio, Finland; 3 National Institute for Health and Welfare (THL), PO Box 30, Lintulahdenkuja 4, FI-00271 Helsinki, Finland; 4 Nordic School of Public Health, PO Box 12133/Nya Varvet SE-402 42 Göteborg, Sweden

## Abstract

**Background:**

Obstetric anal sphincter rupture (OASR) is a serious complication of delivery, which frequently results in faecal incontinence despite primary repair and has serious implications for women's health. The objective of this study was to assess whether human factors, workload and staffing at night, at weekends and during holidays has an effect on the increasing OASR rates among all singleton vaginal deliveries (n = 514,741) having occurred between 1997 and 2007 in Finland. Women (n = 2,849) with OASR were compared in terms of possible risk factors to women without OASR using stepwise logistic regression analysis.

**Findings:**

In Finland, the increase in OASR rate is striking, from 0.2% in 1997 to 0.9% in 2007. OASR rates varied from 0.49% to 0.58% (≤ 0.001) according to the time of day, and were lowest at night. After adjustment for patient-mix and the use of interventions, the risk of OASR was 11% lower (95% CI 3-18%) at night and 15% lower (95% CI 3-26%) in July - the main holiday month. Only 14% of the increased OASR risk during the day time (8-23.59) was attributable to vacuum assistance and birth weight, whereas the holiday period had no effect.

**Conclusions:**

Decreased OASR rates at night and in July suggest that human factors such as decreased alertness due to fatigue or hospitals' administrative factors such as workload and staffing did not increase the rates of OASR.

## Background

Obstetric anal sphincter rupture (OASR) sustained during vaginal delivery is the most common cause of anal incontinence in women, and it has serious and long-term effects on women's health and quality of life [1]. Occurrences of OASR vary widely not only between countries, being 0.9% in 2007 in Finland [2], 1.8% in England and 2.2% in Germany [3], but also over time. In Finland, the increase in OASR rate is substantial, from 0.2% in 1997 to 0.9% in 2007 [2]. Generally, it has been observed that the highest rates are associated with the greatest use of medical interventions during birth [4] and consequently some interventions and demographic characteristics such as vacuum extraction [5-7], forceps delivery [7,8], mediolateral episiotomy [9,10], midline episiotomy [11,12], primiparity [5,8,13,14], a prolonged second stage of delivery [8,15], occiput posterior presentation [5,13,14] and a high birth weight [5,6,8,13] are well-known risk factors for OASR.

The present study is a follow-on from our previous study, in which we determined the risks of anal rupture from the same population. Our results concerning risk factors confirmed the results of previous studies, however, lateral episiotomy -exclusively used in Finland- was associated with decreased OASR risk among primiparous but included in the risk factors among multiparous women [16].

It has been suggested that adverse events in health care were associated with working conditions such as workload and staffing [17]. In addition, the results of one previous study suggested that risk for OASR was 30% higher during the daytime compared to night-time due to higher use of obstetric interventions [18]. The aim of the present study was to assess whether the hospitals' administrative factors such as workload and staffing (patient-to-midwife/obstetrician, skill-mix) and human factors at night, on weekends and during holidays have an effect on OASR rates among singleton vaginal deliveries. The specific aim of the present study was to analyze whether deliveries during July (the most usual holiday month in Finland), on weekends (Saturday or Sunday) or at night (0-8 h) had higher OASR risks compared with those at other times. Our hypothesis was that there would have been more OASRs during these time periods due to these previous mentioned factors.

## Methods

We analyzed retrospectively the population-based Medical Birth Register (MBR), which is currently compiled by THL (National Institute for Health and Welfare). The MBR includes the clinical records from all obstetric care units in Finland, and it contains information maternal and neonatal birth characteristics and perinatal outcomes (live-born or stillborn infants born after the 22nd week of gestation or weighing 500 g or more). Information on OASR has been collected by the MBR since 2004. For the years 1997-2003, the information was taken from the Hospital Discharge Register and OASR equated to ICD-10 codes O70.2 (3^rd ^degree) and O70.3 (4^th ^degree). The two data sources were linked together on the basis of the mothers' unique personal identification numbers. The degree of OASR was classified according to standard definitions: a third degree rupture involves the external anal sphincter and a fourth degree rupture affects both the anal sphincter and anorectal mucosa [1]. In all analyses, data on third and fourth degree obstetric anal sphincter ruptures were pooled.

THL gave the necessary authorization for the use of sensitive health register data in scientific research, as required by national data protection legislation. We used only anonymised data, and thus we did not need the informed consent of the registered individuals.

Statistical differences between the subjects and the reference population were evaluated by using Chi Square tests. Values of *p *≤ 0.05 were considered statistically significant. Logistic regression analysis using a forward elimination procedure was done to examine the association between maternal demographic characteristics (maternal age), birth characteristics (mode of delivery, induction, amniotomy, oxytocin, episiotomy, occiput posterior presentation, epidural analgesia, spinal analgesia, paracervical block, and length of the active second stage of birth), perinatal outcomes (birth weight) and time of birth (month, day of the week, time of day) as independent variables and occurrence of OASR as the dependent variable. Furthermore, in order to examine whether obstetric interventions (vacuum assistance, epidural analgesia, and episiotomy) and background characteristics (maternal age, birth weight) contributed to the risk of OASR during day time and non-holiday time, we estimated the contribution of each of these factors by using logistic regression. The risk of anal rupture in each time factors was adjusted by maternal age and parity in Model B. Each intervention or background characteristics were added separately to the model B, and the contribution of each factor was measured by the percentage reduction in the odds ratio of OASR compared to the Model B (OR Model B - OR Model C/D/E/F)/(OR Model B - 1) [19]. Analyses were performed using SPSS for Windows 16.0.

The mode of delivery was recorded as normal vaginal birth, breech, forceps or vacuum-assisted deliveries. In addition, there were three variables derived from the time of birth: month, day of the week, and time of day. In the univariate analyses time of day was separated into office hours (08.00-15.59), and non-office hours (16.00-23.59 and 00.00-07.59; Additional file 1). In the multivariate analyses, months were separated into July vs. all other months (pooled). July is the most usual holiday month in Finland, and this may result in lower staff competence during this period. Furthermore, days of the week were recorded as weekend (Saturday and Sunday) vs. other days (pooled). Time of day was recorded as night (00.00-07.59) vs. day (08.00-23.59). All continuous variables were classified as categorical variables, for which 95% confidence intervals were calculated in addition to the mean values.

## Results

Our data included all 514,741 women (217,778 primiparous and 296,963 multiparous) in Finland who gave birth to singleton babies vaginally between 1997 and 2007. Of the childbearing women, 2,849 suffered from OASRs (0.6%).

Additional file 1 shows that OASR rates varied significantly (≤ 0.001) from 0.49% at night to 0.58% during day time. Differences between the months and weekdays were not significant. Episiotomy rates were up to 2.1% higher on weekends and 0.9-2.5% lower at night. Further, proportion of over 4000 grams weighted infants were up to 1.8% lower on weekends and up to 1.3% lower at night (p ≤ 0.001).

Table 1 shows the results of multivariate analyses, which confirmed the result of the univariate analyses, and indeed the risk of OASR was 11% lower (95% CI 3-18%, p = 0.01) during the night. In addition, after adjustment, the risk of OASR appeared to be 15% (95% CI 3-26%, p = 0.02) lower in July compared to non-holiday time. Other independent risk factors for OASR included forceps delivery, vacuum assistance, infants weighing over 4,000 grams at birth, episiotomy, epidural analgesia, and augmentation with oxytocin.

**Table 1:** Unadjusted/Adjusted Odds Ratios (OR) of OASR among vaginally delivered women (n = 514,741)

DELIVERY INTERVENTION/ CHARASTERISTICS	Unadjusted OR (95% CI)	Adjusted OR (95% CI)	Adjusted p-value
*Mode of delivery*			
Normal vaginal birth		1	
Forceps	9.48 (6.18-14.55)	22.55 (9.05-56.22)	≤ 0.001
Vacuum assistance	5.40 (4.98-5.86)	7.37 (6.26-8.67)	≤ 0.001

Episiotomy	2.54 (2.35-2.73)	1.95 (1.77-2.13)	≤ 0.001

Episiotomy and vaginal delivery		1	
Episiotomy and forceps	3.02 (2.41-3.80)	0.21 (0.07-0.59)	0.003
Episiotomy and vacuum assistance	2.34 (2.24-2.44)	0.40 (0.33-0.49)	≤ 0.001

*Maternal age (years)*			
≤ 19		1	
20-29	1.29 (1.02-1.64)	1.39 (1.09-1.77)	0.008
30-39	1.18 (0.92-1.30)	1.37 (1.07-1.74)	0.01
≥ 40	0.83 (0.59-1.17)	1.01 (0.71-1.44)	0.95

*Birth weight (g)*			
≤ 2999		1	
3000-3499	1.69 (1.42-2.02)	1.66 (1.40-1.98)	≤ 0.001
3500-3999	2.40 (2.02-2.84)	2.36 (2.00-2.80)	≤ 0.001
≥ 4000	2.87 (2.72-3.86)	3.22 (2.70-3.83)	≤ 0.001

Amniotomy	0.95 (0.89-1.03)	0.91 (0.84-0.98)	0.01

Augmentation with oxytocin	1.89 (1.75-2.04)	1.12 (1.07-1.27)	≤ 0.001

Epidural analgesia	1.96 (1.82-2.11)	1.37 (1.26-1.49)	≤ 0.001

*Spinal analgesia	0.68 (0.54-0.85)	0.77 (0.61-0.96)	0.02

Nitrous oxide gas	1.03 (0.96-1.11)	0.87 (0.80-0.93)	≤ 0.001

*Occiput posterior presentation	7.95 (5.89-10.74)	2.07 (1.52-2.81)	≤ 0.001

*Time of day*			
8-23.59		1	
00.00-7.59	0.85 (0.78-0.92)	0.89 (0.82-0.97)	0.01

*Months*			
All months excluding July		1	
July	0.86 (0.75-0.98)	0.85 (0.74-0.97)	0.02

OR adjusted for mode of delivery, induction, amniotomy, oxytocin, episiotomy, occiput posterior presentation, epidural analgesia, spinal analgesia, paracervical block, maternal age, length of the active second stage of birth, and birth weight) and time of birth (month, day of the week, time).

(*adjusted 2004-2007)

Furthermore, we measured the contribution of vacuum assistance, epidural analgesia, episiotomy, and birth weight to the increased OASR risk during day time and non-holiday time (Additional file 2). Accordingly, 7.1% of the OASR risk during the day could be explained by vacuum assistance. Overall, it appeared that only approximately 14% of OASR risk during the day could be explained by the use of vacuum assistance and by birth weight; the reason for 86% of the occurrences was unknown. During non-holiday time use of interventions or birth weight did not explain the increased OASR risk, as shown in Additional file 2.

## Discussion

There are some strengths and possible bias in this kind of register-based studies. The most important strength was that the register-based data offered a comprehensive picture of OASR risks among the total vaginally delivered population during the study period, and thus there were no selection bias. Data concerning caesarean sections were excluded from the final analysis since women delivered by Cesarean do not have OASRs. In addition, multiple deliveries having a very different risk profile from singletons were also excluded. These exclusions may have had an impact on the results, since there may have been treatment differences in these high risk groups at night and in holiday periods. Therefore the present results can only be generalized for singleton, vaginal deliveries. Possible bias in our study could also result from the fact that this kind of register-based study might include errors and missing values because the data have been collected for administrative purposes. However, the present data were a subset of the national, population-based Medical Birth Register, which has excellent coverage and is of high quality [20,21]. This is especially relevant to incidents that result in surgical repair with specific codes of diagnosis, requiring extra days of hospital care. However, some demographic characteristics and outcomes such as spinal analgesia (marked with * in the Tables) were not collected for the whole study period, and therefore we could only incorporate these in the multivariate analyses for a limited time span, i.e. 2004 onwards. The information on anal ruptures was not available in the MBR before year 2004, but the data were taken from Hospital Discharge Register. Also this register in mandatory for all hospitals and its completeness and quality has been shown to be high [22]. In 2006-2007, for example, it covered 95% of OASRs registered in the MBR.

OASRs are consequences of a complex interplay of maternal (age), fetal (birth weight), human (clinical skills), institutional (policy relating to interventions), and administrative (staffing, workload) factors. This probably applies even to those OASRs that were deemed to be caused by a single, simple factor such as fetal macrosomia. This study concentrated on the human and administrative factors rather than the maternal, fetal and institutional ones, by evaluating OASR rates at night and on weekends and holidays. The synergistic effect caused by maternal, fetal and institutional factors was taken into account in the multivariate analysis.

Based only on the risk of exposure to OASR, the safest time to give birth between 1997 and 2007 in Finland was in July or at night. Herein, we report significantly (p ≤ 0.001) lower OASR rates during night time. After adjustment for patient-mix or use of interventions, the risk of OASR was shown to be 11% lower during night time and 15% lower in July. We expected the opposite result because during holidays the competence of the professionals on duty might be lower due to temporary staff, and during emergency duties (weekends and nights) fewer professionals especially obstetricians are responsible for the service. In addition, particularly at night, fatigue might have an influence on maternal outcomes. Accordingly, it may be speculated that the differences can partly be explained by treatment policy, since during emergency duties and in July obstetricians were more willing to allow women to delivery naturally without intervention. For example, use of vacuum assistance was 0.8-1.3% lower during the night time, and in July the rate of induction was among the lowest in comparison with other months. Consequently, our results somewhat confirmed those of one previous study suggesting that higher OASR rates were associated with higher use of obstetric interventions [18]. Lower OASR rate in July could not be explained by higher cesarean section rate, which was among the lowest in comparison with other months, as shown in Additional file 1. Further, the OASR rate in April (0.49%) was almost as low as in July, and we suggest that it may be to some extent due to Eastern, which increased the number of emergency duties. However, our primary aim was to analyze whether deliveries during July, which is the most popular holiday month in Finland, had higher OASR risks, and therefore we did not perform further analysis concerning lower OASR rate in April. If women had received the same level of care irrespective of the month and if the risk of OASRs had been equal, the total number of OASRs in the eleven-year study period would have decreased by 374 (-13%). Correspondingly, if the same risks had been present during the night and the day, the total number of OASRs would have been 329 lower (-12%).

We believe that the general applicability of the current results is likely to be high for similar health care systems with free access to antenatal and obstetric services. The results are probably less applicable to countries in which the majority of antenatal care and deliveries are given in private healthcare facilities. There may be significant differences in teaching status, staff skills and experience and the use of interventions between these two systems. In Finland, the service is provided equally by trainees and senior doctors also in holiday periods with trainees being on call at hospital and senior doctors at home. Thus, it was not necessary to control for the level of supervision or training. However, national data on staffing patterns of obstetricians and midwifes do not exist, and therefore our conclusions about the OASR rates with respect to seasonal patterns and different times of the day are only speculative reflections about the competence of the staff and the role of human errors. Further, since the adverse event itself is not simply the direct harm associated with OASRs but also the consequences such as undesirable outcomes following repair, all aspects should be considered before drawing any firm conclusions about the safest time to give birth, since the inexperience of staff is likely to be expressed in all aspects of their work.

## Competing interests

The authors declare that they have no competing interests.

## Authors' contributions

All authors (SR, KV-J, MG, and SH) participated in designing the study. SR managed the dataset and performed statistical analyses. KV-J, MG, and SH gave advice regarding the statistical analyses. All authors contributed to the interpretation of the results, as well as to the writing and editing of the manuscript.

## Supplementary Material

Additional file 1 **Rates (%) of episiotomy, OASR, and vacuum assistance and proportions (%) of over 4000 grams weighted infants during months, weekdays and time of day in vaginal delivered women (n = 514,741) with singleton pregnancy during 1997-2007 in Finland (Chi Square test)**. Table S1 in landscape orientation. Click here for file

Additional file 2 **Odds ratios (OR) for OASR by each time factor before and after adjustment for interventions**. Table S3 in landscape orientation. Click here for file
