# Supplementary material for: The role of nocturnal delivery and delivery during the holiday period in Finland on obstetric anal sphincter rupture rates- a population based observational study
Source: BMC Res Notes. 2010 Feb 5;3:32. doi: 10.1186/1756-0500-3-32 (PMC2828462; doi:10.1186/1756-0500-3-32)
Supplement: Additional file 1 — Rates (%) of episiotomy, OASR, and vacuum assistance and proportions (%) of over 4000 grams weighted infants during months, weekdays and time of day in vaginal delivered women (n = 514,741) with singleton pregnancy during 1997-2007 in Finland (Chi Square test). Table S1 in landscape orientation. [file 1756-0500-3-32-S1.RTF]

Table 1. Rates (%) of episiotomy, OASR, and vacuum assistance and proportions (%) of over 4000 grams weighted infants during months, weekdays and time of day in vaginal delivered women (n=514,741) with singleton pregnancy during 1997-2007 in Finland (Chi Square test). 

Time factor	OASR %	p value	Episiotomy %	p value	Vacuum assistance %	p value	>4000 g birth weight %	p value	Induction
%	p
value		*Cesarean section %
(n=96,774)	p
value	
Month
January
February
March
April
May
June 
July
August
September
October
November
December	
0.56
0.58
0.57
0.49
0.55
0.58
0.48
0.54
0.55
0.62
0.53
0.60	
0.17	
36.9
36.5
35.3
35.3
35.5
36.6
36.3
35.7
35.5
35.6
35.2
35.3	
≤0.001	
7.2
7.6
7.4
7.3
7.6
7.8
7.8
7.8
7.6
7.8
7.7
7.8
	
≤0.001	
19.3
19.1
18.9
19.2
18.5
18.1
18.5
18.4
18.7
19.1
18.3
18.3	
≤0.001	
16.4
16.7
16.1
16.0
15.7
15.5
15.6
15.9
16.3
16.4
16.8
16.3	
≤0.001		
16.0
15.9
15.5
15.5
15.7
15.8
15.8
15.8
15.8
16.2
16.2
16.2	
≤0.001

	
Weekday
Monday
Tuesday
Wednesday
Thursday
Friday
Saturday
Sunday	
0.55
0.57
0.55
0.57
0.57
0.52
0.54
	
0.89	
34.8
35.4
35.7
35.8
35.7
36.9
36.5
	
≤0.001	
7.4
7.7
7.6
7.7
7.8
7.5
7.7
	
0.06	
18.9
19.3
18.9
19.1
18.9
18.0
17.5
	
≤0.001	
15.1
18.3
18.3
18.6
19.2
12.9
8.8
	
≤0.001
		
17.1
18.0
17.3
18.3
18.0
9.9
9.6
	
≤0.001
	
Time of day
0-7.59
8-15.59
16-23.59	
0.49
0.58
0.58	
≤0.001	
34.7
35.6
37.2
	
≤0.001	
6.9
7.7
8.2
	
≤0.001	
17.8
18.9
19.2
	
≤0.001	
10.3
11.5
25.8	
≤0.001		
7.9
28.2
10.6
	
≤0.001	
Pooled	0.55		35.8		7.6		18.7		16.1			15.8
		
*Cesarean sections rates are also given during the same period of time
