# Supplementary material for: The role of nocturnal delivery and delivery during the holiday period in Finland on obstetric anal sphincter rupture rates- a population based observational study
Source: BMC Res Notes. 2010 Feb 5;3:32. doi: 10.1186/1756-0500-3-32 (PMC2828462; doi:10.1186/1756-0500-3-32)
Supplement: Additional file 2 — Odds ratios (OR) for OASR by each time factor before and after adjustment for interventions. Table S3 in landscape orientation. [file 1756-0500-3-32-S2.RTF]

Table 3. Odds ratios (OR) for OASR by each time factor before and after adjustment for interventions 
	Model A	Model B	Model C	Model D	Model E	Model F	
	OR (95 % CI)	OR (95 % CI)	OR (95 % CI)	Diff. with
B (%)*	OR (95 % CI)	Diff. with
B (%)*	OR (95 % CI)	Diff. with
B (%)*	OR (95 % CI)	Diff. with
B (%)*	
Day time
 (8-23.59)	1.18 (1.09-1.29)	1.14 (1.05-1.25)	1.13 (1.04-1.23)	7.1	1.14 (1.05-1.24)	0	1.14 (1.05-1.25)	0	1.13 (1.04-1.23)	7.1
	
Months 
excluding July	1.17 (1.01-1.34)		1.17 (1.02-1.34)		1.17 (1.02-1.35)		0	1.17 (1.02-1.34)		0	1.17 (1.02-1.34)	0	1.17 (1.02-1.34)	0	

*(The contribution of each factor was measured by the percentage reduction in the odds ratio of OASR compared to Model B by using formula (OR Model B – OR Model C/D/E/F) / (OR Model B – 1)
Model A= Adjusted for day time or months excluding July
Model B= Adjusted for day time or months excluding July, maternal age and parity (primi- or multiparous)
Model C= Adjusted for day time or months excluding July, maternal age, parity (primi- or multiparous), and vacuum assistance
Model D= Adjusted for day time or months excluding July, maternal age, parity (primi- or multiparous), and epidural analgesia
Model E= Adjusted for day time or months excluding July, maternal age, parity (primi- or multiparous), and episiotomy
Model F= Adjusted for day time or months excluding July, maternal age, parity (primi- or multiparous), and birth weight
